# The combination of low‐intensity resistance exercise and electrical muscle stimulation effectively enhances executive function in men

**DOI:** 10.1111/cpf.70075

**Published:** 2026-06-23

**Authors:** Kento Dora, Su Yang, I. Wayan Yuuki, Kousei Tachi, Kaito Hashimoto, Teppei Matsumura, Naokazu Miyamoto, Takeshi Hashimoto

**Affiliations:** ^1^ Faculty of Sport and Health Science Ritsumeikan University Shiga Japan; ^2^ Department of Biomedical Engineering Toyo University Saitama Japan; ^3^ Research Organization of Science and Technology Ritsumeikan University Shiga Japan; ^4^ Research Fellow of Japan Society for the Promotion of Science Tokyo Japan; ^5^ Faculty of Health and Sports Science Juntendo University Chiba Japan; ^6^ Faculty of Sport Sciences Waseda University Saitama Japan

**Keywords:** cognitive function, exercise, inhibitory control, lactate, muscle contraction

## Abstract

**Objectives:**

Resistance exercise enhances executive function (EF) more effectively at moderate or higher intensities than at low intensity resistance exercise (LRE), probably with increased neural activity and lactate production to meet brain energy demands. Given that LRE remains widely applicable with less perceived exertion, and electrical muscle stimulation (EMS) increases lactate production and neural activation, we investigated whether combining LRE with EMS (LRE‐EMS) effectively enhances EF, without increasing perceived exertion.

**Design:**

A crossover randomized study.

**Methods:**

Seventeen young men participated in a crossover randomized study to assess the cognitive effects of LRE alone, EMS alone, and LRE‐EMS. The LRE protocol involved knee extensions at 40% one‐repetition maximum for 4 sets of 10 repetitions. During the EMS condition, the participants remained seated for 260 s to match the duration of the other conditions, with EMS applied to the lower limb muscles. Subjective perceptions of exertion and fatigue during exercise were recorded.

**Results:**

IC and lactate were measured at baseline, immediately postexercise, and 15 min postexercise. The LRE condition did not significantly increase IC (*p* = 0.100), whereas LRE‐EMS resulted in significant IC improvements immediately and 15 min postexercise (both *p* < 0.001), with greater lactate increases compared to LRE and EMS. EMS demonstrated significant IC improvement at 15 min postexercise (*p* = 0.022).

**Conclusions:**

Perceived exertion and fatigue were greater with LRE‐EMS compared with LRE and EMS. These findings suggest that LRE combined with EMS and EMS alone are effective strategies for cognitive improvement, but increased perceived exertion still exists.

**Abbreviations**
1‐RM: One‐repetition maximum
ANOVA: Analysis of variance
CR‐10: Category ratio scale
CWST: Color‐word Stroop task
EMS: Electrical muscle stimulation
FAS: Felt arousal scale
HR: Heart rate
IC: Inhibitory control
LRE: Low intensity resistance exercise
LRE‐EMS: Combining low intensity resistance exercise with electrical muscle stimulation
RPE: Rating of perceived exertion
VAS: Visual analog scale

## 1 INTRODUCTION

Executive function (EF), as a higher‐order cognitive function, is essential for everyday functioning and quality of life (Gamage et al., 2018). Growing evidence suggests that acute physical exercise, such as aerobic (Voss et al., 2020) and resistance exercise (Chang et al., 2012; Dora et al., 2021; Tsukamoto et al., 2017), has the potential to improve EF performance. As aging leads to a progressive decline in muscle mass, which negatively impacts quality of life (Trombetti et al., 2016) and EF ability (Tessier et al., 2022), exercise, particularly resistance exercise, has emerged as an essential intervention. This form of exercise not only helps preserve or increase skeletal muscle mass (American College of Sports Medicine, 2009) but also supports cognitive health (Northey et al., 2018; Zhang et al., 2023), highlighting its importance in public health initiatives.

Despite these advantages, determining an ideal exercise regimen that optimally benefits cognitive function across diverse populations remains challenging due to variables such as exercise type, intensity, and individual characteristics (Herold et al., 2019). In terms of exercise intensity, compared with low intensity resistance exercise (LRE), high‐intensity localized resistance exercise has been shown to result in more pronounced improvements in inhibitory control (IC) (Tsukamoto et al., 2017). IC is a fundamental component of executive function, which is an essential aspect of cognitive function. Moreover, IC is critical for suppressing inappropriate actions (Coxon et al., 2007). Although the precise mechanisms underlying this advantage are unknown, they may be partly attributed to augmented neural activation (Hyodo et al., 2012; Yanagisawa et al., 2010), which becomes more pronounced with exercise intensity (Endo et al., 2013). Additionally, lactate, a metabolite generated by contracting muscles, energetically supports neuronal activation and enhances exercise‐induced cognitive improvement (Hashimoto et al., 2018, 2021). Consequently, high‐intensity resistance exercise is regarded as an effective approach for enhancing cognitive function owing to its greater impact on neural activity and increased lactate production from contracting skeletal muscles to meet the energy demands of the brain.

Nevertheless, moderate‐ to high‐intensity resistance exercise (>70% one‐repetition maximum [1‐RM]) decreases vascular function (Miyachi, 2013) and is not advisable for individuals with joint disorders and chronic diseases owing to increased risks of adverse outcomes compared with LRE (Messier et al., 2021; Williams et al., 2007). To address these concerns, Dora et al. proposed a slow movement and tonic force generation protocol that demonstrated superior performance over conventional LRE in promoting lactate production and enhancing IC (Dora et al., 2021). However, participants reported higher ratings of perceived exertion (RPE) under this protocol, a factor that could reduce long‐term adherence, particularly among older adults (Hurley et al., 2018; Jack et al., 2010). Therefore, developing resistance exercise protocols that effectively enhance cognitive function without increasing subjective effort is essential.

Based on this background, adding electrical muscle stimulation (EMS) to LRE, instead of using a slow movement and tonic force generation protocol, may be worth exploring. EMS is increasingly recognized as a promising alternative exercise modality, particularly for populations with limited physical activity, such as inactive individuals (Pano‐Rodriguez et al., 2019), with evidence indicating its ability to enhance cognitive performance following acute interventions (Chaney et al., 2024; Descollonges et al., 2024a, 2024b). Importantly, EMS enhances neural activity by promoting sustained neuroplastic adaptations in central motor pathways (Carson and Buick, 2021) and increasing the blood lactate concentration (Miyamoto et al., 2015, 2018). Furthermore, adding EMS during exercise enhances physiological responses without increasing perceived exertion. For example, studies have revealed that adding EMS to aerobic exercise increases oxygen consumption and blood lactate levels without causing a concurrent increase in subjective effort (Watanabe et al., 2021). Similarly, EMS has been shown to achieve comparable cerebral oxygenation responses to voluntary exercise despite reduced voluntary muscle activation due to external assistance (Descollonges et al., 2026). In line with this, several studies have investigated the effects of adding EMS to voluntary aerobic exercise on cognitive performance. However, the findings remain inconsistent, with some studies reporting additional improvements in cognitive function when EMS is superimposed onto voluntary exercise (Descollonges et al., 2025, 2026), whereas others have found no such effects (Ando et al., 2024a, 2024b). Notably, the effects of combining EMS with resistance exercise on cognitive function have not yet been investigated.

Overall, we hypothesized that incorporating EMS into LRE could effectively enhance cognitive function without increasing perceived exertion. To evaluate this hypothesis, we compared the acute effects of a combined LRE and EMS protocol (LRE‐EMS) with those of LRE on improving IC. Furthermore, to investigate the independent effects of EMS under the specific parameters utilized in this study, we included an additional condition where EMS was applied in isolation.

## 2 METHODS

### 2.1 Participants

Prior to this study, we calculated the required sample size utilizing an effect size of 0.42, an α‐level of 0.05, and a β‐level of 0.2 (80% power) on the basis of the data (i.e., the reverse‐Stroop interference score) from our previous study (Dora et al., 2021). The calculated necessary number of subjects was 12. Considering the possibility of participant drop‐out and to ensure statistical power and sensitivity, seventeen healthy young men (age: 23 ± 1 years, height: 174.2 ± 4.8 cm, weight: 67.9 ± 6.6 kg, 1‐RM for bilateral knee extension: 120.4 ± 21.2 kg) were recruited for this study. None of the participants had a history of neurological, cardiovascular, or pulmonary disorders; color blindness; or visual impairments. The participants were instructed to abstain from strenuous physical activity, alcohol, and caffeine consumption for 24 h prior to the start of the experimental procedure. Additionally, they adhered to an overnight fast, with water permitted, for 12 h before the experimental session. No participant was taking medication that could influence cognitive function. A minimum interval of 2 days was maintained between experimental conditions based on previous study (Ando et al., 2024b), to minimize potential carryover effects such as muscle fatigue, and residual physiological responses. This study was conducted in compliance with the *Declaration of Helsinki*, and all procedures were approved by the Ethics Committee of Ritsumeikan University (BKC‐LSMH‐2023‐100‐1).

### 2.2 Experimental procedure

Before the day of the experiment, all the participants practiced the color‐word Stroop task (CWST) to measure their IC until they achieved consistent scores. The subjects subsequently underwent 1‐RM measurements of bilateral knee extension and EMS reference intensity measurements used for the EMS condition and the LRE‐EMS condition. Afterward, they underwent experiments for 3 days (i.e., conditions) with a wash‐out period of at least 2 days.

**Figure 1:**
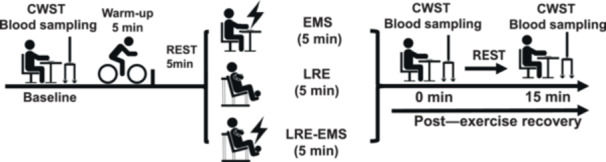
Overview of the experimental protocol.

A counterbalanced crossover randomized design was employed to randomly assign participants to three conditions. On the experimental days (Figure 1), upon participant arrival, the participants practiced the CWST again to minimize learning effects (Tsukamoto et al., 2017). Following their arrival, the participants were seated and maintained in a resting state for 10 min. Following the resting period, blood samples were collected from the fingertip of the hand not used for the cognitive task (index, middle, or ring finger), and measurements of blood parameters (blood glucose levels and blood lactate concentrations) were taken. Afterward, baseline IC and psychological conditions were assessed.

The participants performed a 5‐min warm‐up on a bicycle ergometer (Life Fitness 95 C Inspire Upright Bike, Life Fitness, Schiller Park, IL, United States) at 50 watts. After completing the warm‐up and a subsequent 5‐min rest, they participated in three conditions: 1) EMS condition, 2) LRE condition, and 3) LRE‐EMS condition. In conditions involving voluntary exercise (i.e., LRE and LRE + EMS), perceived exertion and heart rate (HR) were measured and recorded immediately after each set. In the EMS‐only condition, HR was measured at the corresponding time points; however, perceived exertion was not assessed, as no voluntary movement was performed. In the EMS condition, participants sat on the bilateral knee extensions on a leg extension machine for 260 s (matching the duration of the LRE) without performing any active movement while receiving electrical muscle stimulation. In the LRE‐EMS condition, participants performed the same training as in the LRE condition while receiving electrical stimulation. Blood samples and measurements of blood parameters, including blood glucose levels and blood lactate concentrations, were collected at two time points: immediately after the intervention (i.e., post‐EX 0) and 15 min postintervention (i.e., post‐EX 15). In addition, IC and psychological conditions were assessed at the same time points, with the 15‐min postintervention assessment aimed at investigating the sustainable effects of postexercise IC improvements.

### 2.3 Exercise protocols

The LRE was standardized at 40% (Tsukamoto et al., 2017) of the participants' 1‐RM (see below). The protocol involved performing bilateral knee extensions on a leg extension machine (Life Fitness; Schiller Park, IL, United States) for four sets of ten repetitions, with a tempo of 1 s for the concentric phase and 1 s for the eccentric phase (Tsukamoto et al., 2017). A 1‐min rest interval (Dora et al., 2021) was implemented between sets, resulting in a total protocol duration of 260 s.

In this study, we used the latest device (ESPURGE, Ito Ultrashortwave Co., Ltd., Saitama, Japan) from the same company that manufactured the device used in prior research (Miyamoto et al., 2011). Electrical stimulation was applied to the quadriceps muscles. In accordance with a previous study (Miyamoto et al., 2011), one electrode was attached near the patella on the quadriceps, whereas the other was placed on the upper half of the quadriceps muscle belly, closer to the greater trochanter. The intensity of the electrical current was based on the reference intensity measured on the first day of the experiment. The electrical muscle stimulation parameters were set based on prior studies demonstrating that EMS alone can elevate blood lactate levels and that, when superimposed onto voluntary exercise, it further enhances lactate responses (Miyamoto et al., 2018; Watanabe et al., 2021). In addition, as higher stimulation frequencies induce complete or incomplete tetanic contractions that may interfere with voluntary movement, a low frequency was selected (Watanabe et al., 2014). Specifically, a frequency of 4 Hz and a pulse width of 0.25 ms were applied.

In the EMS condition, participants remained in a fully passive seated position on a training machine without performing any voluntary contraction against resistance. In the LRE‐EMS condition, continuous EMS was applied during the 260‐second duration of the LRE protocol. The EMS intensity was standardized and kept consistent between the EMS‐only and LRE + EMS conditions. The LRE intensity was 47.6 ± 8.7 kg (mean ± SD), and the EMS intensity was 64 ± 9 mA (mean ± SD).

### 2.4 Measurements

#### 2.4.1 One‐repetition maximum

The participant's 1‐RM was determined during the first visit via bilateral knee extension exercise, following the protocol established in a prior study (Dora et al., 2021). The 1‐RM value was used to calculate the load for both the LRE and LRE‐EMS conditions. The 1‐RM trial began with 10‐kg increments until reaching approximately 60%–80% of the participant's perceived maximum. Thereafter, the load gradually increased by 1–5 kg until lift failure occurred, which was defined as the point at which the participant could no longer maintain proper form or fully lift the weight. The highest successfully lifted load was recorded as the 1‐RM.

#### 2.4.2 Intensity of EMS

In this study, a low‐frequency EMS device (ESPURGE, Ito Ultrashortwave Co., Ltd., Saitama, Japan) was used according to the manufacturer's instructions. To ensure the continuous monitoring of participants' conditions during incremental increases in EMS intensity, the intensity level for each participant was determined using a 12‐level incremental intensity EMS trial (Watanabe et al., 2023). During the trial, the intensity was increased in increments of 10 mA per level until reaching 40 mA. Beyond this threshold, owing to heightened discomfort reported by the participants, the increase was reduced to 5 mA per level, with a maximum intensity of 80 mA, resulting in a total of 12 levels. Upon reaching a new level, further increases in EMS intensity were temporarily halted, and the participant's condition was reassessed. The level of discomfort induced by electrical stimulation was evaluated using a visual analog scale (VAS). If the participant reported discomfort that exceeded half of the scale and approached “very uncomfortable” (the upper limit of the VAS), they were instructed to say “stop” when they felt that they had reached their limit. The researcher observed the participants closely and proceeded with caution. When the participant said “stop” or the maximum intensity of the device was reached, the device was immediately powered off, and the intensity at that point was recorded. If the participant said “stop,” the experiment proceeded using an intensity of 5 mA lower than the limit they had indicated. This value was used as the reference intensity for later use. After a 5‐min rest, the EMS was applied at the reference intensity, and the participant performed knee extension exercises at 40% of their 1‐RM. If the participant was unable to complete the knee extensions in sync with the rhythm (1 s for the concentric phase and 1 s for the eccentric phase) due to electrical stimulation, the intensity was gradually reduced by 5 mA until the participant was able to perform the movement smoothly. The final intensity was recorded and used as the EMS reference intensity for the EMS condition and the LRE‐EMS condition.

#### 2.4.3 Inhibitory control

The CWST was used to assess IC, following the methodology outlined in a prior study (Dora et al., 2021). In summary, 24 stimulus words representing four color names (red, blue, green, and yellow, written in Japanese characters) were randomly displayed for each task. The participants completed each of the three CWST subcategories—congruent, neutral, and incongruent—three times, resulting in a total of nine trials per test. The task conditions included congruent (e.g., the word “red” displayed in red font), neutral (e.g., the word “red” displayed in black font), and incongruent (e.g., the word “red” displayed in blue, yellow, or green font) tasks. The participants were instructed to press the color‐labeled key corresponding to the meaning of the word. The total reaction time for all 24 stimulus words and response accuracy were recorded for analysis.

IC was evaluated using the reverse‐Stroop interference score, which was calculated as the difference between the average reaction times for the neutral and incongruent tasks (Ikeda et al., 2010). The reverse‐Stroop interference score was expressed as follows: [(reaction time on the incongruent task – reaction time on the neutral task)/reaction time on the neutral task × 100] (Ikeda et al., 2010).

Participants performed practice trials as a cognitive task familiarization, completing each CWST condition at least 10 times before the day of the experiment. This approach was based on our previous study (Tsukamoto et al., 2017), which suggested that learning effects were minimized.

#### 2.4.4 Blood parameters and cardiovascular response

Blood glucose and lactate levels were measured using a glucose analyzer (Glucocard G Black, Arkray, Inc., Kyoto, Japan) and a lactate analyzer (Lactate Pro 2, Arkray, Inc., Kyoto, Japan), respectively. Heart rate (HR) fluctuations during the experiment were monitored using a heart rate sensor (Polar Electro Japan RS400, Tokyo, Japan). With respect to HR, due to equipment malfunction, data from three participants were unavailable; therefore, data from the remaining 14 participants were included in the analysis.

#### 2.4.5 Psychological conditions

Perceived exertion during exercise was assessed using Borg's RPE scale, which ranges from 6 (no exertion) to 20 (maximal exertion) (Borg, 1982). Additionally, leg pain during exercise was evaluated using the Borg Category Ratio Scale (CR‐10), which ranges from 0 (nothing at all) to 10 (very, very strong) (Borg, 1982). Participants were instructed to rate their perceived exertion and leg pain based on the sensations experienced during the immediately preceding exercise set (i.e., “Please indicate the intensity of the exercise you just performed” and “Please indicate the level of pain in your exercising leg”). Perceived exertion and leg pain scores were recorded immediately after each set, and the mean values across all four sets were calculated for subsequent analysis.

Because exercise‐induced increases in arousal have been associated with post‐exercise improvements in IC (Byun et al., 2014), arousal levels were measured immediately following the completion of the CWST using the Felt Arousal Scale (FAS), a 6‐point single‐item scale ranging from 1 (low arousal) to 6 (high arousal) (Svebak and Murgatroyd, 1985). In addition, a VAS was employed to evaluate psychological states such as mental fatigue, concentration ability, and motivation. Each VAS ranged from 0 mm (not at all) to 100 mm (extremely), and participants marked the VAS to indicate their psychological state during the CWST (Dora et al., 2021).

### 2.5 Statistics

All the data are presented as the means ± SDs when the normality of the data distribution was confirmed using the Shapiro–Wilk test. For data not normally distributed, the results are expressed as medians (IQRs). Statistical analyses were performed using two‐way repeated‐measures analysis of variance (ANOVA) with condition and time as factors, provided that the normality assumption was satisfied. If the assumption of sphericity was violated, Greenhouse–Geisser corrections were applied. *Post hoc* analyses for specific differences between time points were conducted using paired *t* tests. After confirming no baseline differences between conditions, we determined the appropriate statistical test based on normality at each condition level. Specifically, if all time points within a given condition satisfied normality assumption, one‐way repeated‐measures ANOVA were applied. Conversely, for conditions containing any time points that violated normality assumptions, Friedman tests were used to assess time effects within that condition. If significant main effects were detected, pairwise comparisons were performed using paired t tests or Wilcoxon signed‐rank tests, as appropriate. Additionally, for significant main effects identified using one‐way repeated‐measures ANOVA or Friedman tests, absolute changes in variables from baseline to postexercise (i.e., post‐EX 0 ‐ baseline, post‐EX 15 ‐ baseline) were also analyzed using the same statistical framework, followed by condition‐level comparisons using paired *t* tests or Wilcoxon signed‐rank tests. HR, perceived exertion and leg pain during exercise were compared using paired *t* tests or Wilcoxon signed‐rank tests. For all multiple comparisons, Bonferroni correction was applied. Statistical significance was set at *p* < 0.05. For data with a normal distribution, effect sizes were calculated as Cohen's *d* using the mean and pooled SD, along with 95% confidence intervals, to evaluate the magnitude of differences. The interpretation of Cohen's *d* values followed standard thresholds: small (0.20 ≤ *d* < 0.50), medium (0.50 ≤ *d* < 0.80), and large (0.80 ≤ *d*) (Cohen, 1992). For nonnormal data, effect sizes were calculated as *r*, derived from the *Z* score of the Wilcoxon signed‐rank test. The interpretation of *r* followed similar thresholds: small (0.10 ≤ *r* < 0.30), medium (0.30 ≤ *r* < 0.50), and large (0.50 ≤ *r*) (Cohen, 1992).

All the statistical analyses were conducted using IBM SPSS Statistics software (version 29.0, IBM Corp., Armonk, NY, United States). Regarding the figures, individual, box‐and‐whisker, and raincloud plots were created via JASP software (version 0.18.1.0, University of Amsterdam, Amsterdam, Netherlands).

## 3 RESULTS

For parameters for which normality was not confirmed, no significant differences were found between conditions at baseline (Supplementary Table 1).

### 3.1 Measured variables during exercise sessions

Table 1 presents the mean values of cardiovascular responses and perceived exertion during the LRE‐EMS, LRE, and EMS conditions. The mean heart rate during exercise was greater in the LRE‐EMS condition (*p* < 0.001, *d* = 1.404; Table 1) and the LRE condition (*p* < 0.001, *d* = 1.352) compared to the EMS condition. The mean perceived exertion values during exercise were greater in the LRE‐EMS condition compared to the LRE condition (*p* = 0.003, *r* = 0.716). Similarly, the mean leg pain values during exercise were greater in the LRE‐EMS condition compared with the LRE (*p* = 0.006, *r* = 0.736) and EMS (*p* = 0.018, *r* = 0.672) conditions, whereas no significant differences were observed between the LRE and EMS conditions (*p* = 0.795, *r* = 0.063).

**Table 1:** Mean values of cardiovascular and perceived exertion parameters during LRE, EMS, and LRE‐EMS conditions.

				*p*‐values			
	LRE	EMS	LRE‐EMS	one‐way ANOVA or Friedman test	LRE vs. EMS	LRE vs. LRE‐EMS	EMS vs. LRE‐EMS
Heart rate, bpm	97 ± 12	77 ± 9	99 ± 13	**<0.001**	**<0.001**	0.533	**<0.001**
Rating of perceived exertion, 6‐20	13 (8–15)	N/A	14 (10–17)	N/A	N/A	**0.003**	N/A
Leg discomfort, 0–10	4 (2–6)	4 (1–7)	5 (1–8)	**0.002**	2.385	**0.006**	**0.018**

*Note*: Values are presented as Mean ± SEM. Mean values of measured variables were calculated as the average of all four sets. The *p*‐values shown in the table represent the results of one‐way analysis of time or Friedman test, and the comparison of between conditions by paired *t*‐test or Wilcoxon signed rank test with Bonferroni correction. Significant values are in bold. LRE; low‐intensity resistance exercise; EMS; electrical muscle stimulation; LRE‐EMS; combining LRE with EMS.

### 3.2 Changes in blood metabolites during the experimental session

No significant changes in blood glucose levels were observed under any of the experimental conditions (Table 2). Lactate levels increased significantly immediately after exercise (*p* < 0.001, *r* = 0.879 for LRE; *p* < 0.001, *r* = 0.878 for EMS; *p* < 0.001, *r* = 0.878 for LRE‐EMS) and 15 min postexercise (*p* = 0.003, *r* = 0.800 for LRE; *p* = 0.007, *r* = 0.743 for EMS; *p* = 0.001, *r* = 0.853 for LRE‐EMS) compared with baseline in all three conditions (Table 2). The extent of the increase in lactate from baseline to immediately after exercise was significantly greater in the LRE‐EMS condition than in the LRE condition (*p* = 0.003, *r* = 0.790; Table 3) and the EMS condition (*p* < 0.001, *r* = 0.878). Similarly, the increase from baseline to immediately after exercise was significantly greater in the LRE condition than in the EMS condition (*p* = 0.004, *r* = 0.786). Regarding the change from baseline to 15 min postexercise, the increase was significantly greater in the LRE‐EMS condition than in both the LRE (*p* = 0.004, *d* = 0.909) and EMS conditions (*p* < 0.001, *d* = 1.085), with no significant difference between the LRE and EMS conditions (*p* = 0.186, *d* = 0.463).

**Table 2:** Blood metabolites, cognitive tasks and psychological conditions throughout LRE, EMS, and LRE‐EMS conditions.

				*p*‐values			
	Time points			two‐way ANOVA			one‐way ANOVA or Friedman test
	Baseline	post‐EX 0	post‐EX 15	Condition	Time	Interaction	
***Blood metabolites***							
Glucose (mg/dL)							
LRE	92 ± 11	89 ± 12	93 ± 11				0.075
EMS	90 (79–104)	86 (80–116)	95 (76–112)		N/A		0.476
LRE‐EMS	91 (75–108)	87 (77–120)	91 (59–112)				0.630
Lactate (mM)							
LRE	1.4 (1.0–2.3)	3.6 (2.6–11.3)**	2.0 (1.4–4.9)* ^,^ ††				**<0.001**
EMS	1.3 (0.9–2.5)	2.7 (1.7–4.5)**	2.0 (1.2–3.5)* ^,^ †		N/A		**<0.001**
LRE‐EMS	1.4 (1.0–2.4)	6.3 (3.6–11.3)**	3.5 (1.7–6.2)* ^,^ ††				**<0.001**
***Color‐word Stroop tasks***							
Reaction time (msec)							
Congruent task							
LRE	11705 ± 1620	11459 ± 1892	11649 ± 1751				
EMS	12234 ± 2520	11655 ± 2499	11704 ± 2211	0.593	0.069	0.825	N/A
LRE‐EMS	12175 ± 2223	11642 ± 2349	11822 ± 2460				
Neutral task							
LRE	12545 ± 2142	12371 ± 2135	12299 ± 2165				
EMS	12457 ± 2384	12615 ± 2657	12490 ± 2328	0.904	0.726	0.700	N/A
LRE‐EMS	12311 ± 2046	12581 ± 1977	12502 ± 2420				
Incongruent task			**				
LRE	13499 ± 2464	12995 ± 2370	12714 ± 2235				
EMS	13425 ± 2530	13148 ± 2883	12861 ± 2449	0.755	**<0.001**	0.877	N/A
LRE‐EMS	13402 ± 2412	12786 ± 2413	12697 ± 2922				
Response accuracy (%)							
Congruent task							
LRE	99 (92–100)	97 (86–100)	97 (81–100)				0.071
EMS	96 (86–100)	99 (93–100)	97 (90–100)		N/A		0.108
LRE‐EMS	97 (92–100)	97 (77–100)	99 (94–99)				0.571
Neutral task							
LRE	100 (93–100)	100 (92–100)	99 (93–100)				0.758
EMS	100 (83–100)	99 (93–100)	99 (89–100)		N/A		0.174
LRE‐EMS	99 (88–100)	99 (89–100)	99 (92–100)				0.401
Incongruent task							
LRE	99 (89–100)	97 (86–100)*	99 (81–100)				**0.044**
EMS	99 (92–100)	99 (85–100)	99 (88–100)		N/A		0.643
LRE‐EMS	99 (94–100)	97 (85–100)	99 (88–100)				0.423
***Psychological states***							
Felt arousal scale, 1–6							
Arousal							
LRE	3 (2–4)	4 (2–5)*	3 (2–5)*				**<0.001**
EMS	3 (2–5)	3 (2–5)*	3 (1–5)		N/A		**0.010**
LRE‐EMS	3 (2–4)	4 (2–5)*	3 (1–5)				**0.001**
Visual analog scales, 0–100 mm							
Mental fatigue							
LRE	13 (1–61)	31 (1–67)*	25 (1–81)*				**0.010**
EMS	17 (1–79)	34 (0–83)	22 (0–82)		N/A		0.099
LRE‐EMS	13 (1–72)	34 (0–73)*	36 (0–76)*				**0.002**
Concentrate							
LRE	70 ± 21	71 ± 18	71 ± 16				
EMS	67 ± 21	73 ± 14	71 ± 17	0.650	0.720	0.367	N/A
LRE‐EMS	71 ± 19	68 ± 20	66 ± 22				
Motivation							
LRE	73 ± 18	74 ± 17	76 ± 17				
EMS	75 ± 17	74 ± 17	73 ± 16	0.962	0.772	0.255	N/A
LRE‐EMS	76 ± 18	72 ± 18	74 ± 18				

*Note*: Values are mean ± SD or median (IQR). The *p*‐values shown in the table represent the results of two‐way analysis of variance, one‐way analysis of time or Friedman test, and the letters * and † represent the results of the comparison between time points by paired *t*‐test or Wilcoxon signed rank test with Bonferroni correction. Significant values are in bold. post‐EX 0; immediately after exercise; post‐EX 15; 15‐min at post‐exercise recovery period.

* *p* < 0.05 versus baseline

** *p* < 0.01 versus baseline.

^†^ *p* < 0.05 versus post‐EX 0.

^††^ *p* < 0.01 versus post‐EX 0.

**Table 3:** Comparison of changes in lactate, cognitive tasks, and psychological conditions between LRE, EMS and LRE‐EMS conditions.

	Condition			one‐way ANOVA or Friedman test
	LRE	EMS	LRE‐EMS	*p* value
***Blood metabolites***				
Lactate (mM)				
Δpost‐EX 0 ‐ baseline	2.1 (0.9–9.5)	1.2 (0.4–2.9)*	4.9 (1.7–8.9)* ^,^ ††	**<0.001**
Δpost‐EX 15 ‐ baseline	1.0 ± 0.9	0.5 ± 0.5	2.2 ± 1.4* ^,^ ††	**<0.001**
***Color‐word Stroop tasks***				
Response accuracy (%)				
Incongruent task				
Δpost‐EX 0 ‐ baseline	−1 (−6–1)	0 (−7–4)	0 (−10–4)	0.298
Δpost‐EX 15 ‐ baseline	0 (−8–1)	0 (−4–6)	0 (−7–4)	0.106
Interference scores (%)				
Δpost‐EX 0 ‐ baseline	−2.7 ± 4.6	−3.7 ± 8.5	−7.0 ± 6.2	0.127
Δpost‐EX 15 ‐ baseline	−1.7 (−18.6–10.1)	−3.9 (−24.7–2.5)	−7.0 (−19.1–1)	0.193
***Psychological states***				
Felt arousal scale, 1–6				
Arousal				
Δpost‐EX 0 ‐ baseline	1 (0–2)	0 (0–1)	1 (0–2)	0.310
Δpost‐EX 15 ‐ baseline	1 (−1–2)	1 (−1–2)	1 (−1–1)	0.294
Visual analog scales, 0‐100 mm				
Mental fatigue				
Δpost‐EX 0 ‐ baseline	6 (−5–32)	2 (−7–28)	12 (−11–51)	0.153
Δpost‐EX 15 ‐ baseline	10 (−11–44)	3 (−8–44)	13 (−4–46)	0.072

*Note*: Values are mean ± SD or median (IQR). The *p*‐values shown in the table represent the results of one‐way analysis of time or Friedman test, and the letters * and ^†^ represent the results of the comparison of changes from baseline to each time point (∆) between conditions by paired *t*‐test or Wilcoxon signed rank test with Bonferroni correction. Significant values are in bold. LRE; low‐intensity resistance exercise; EMS; electrical muscle stimulation; LRE‐EMS; combining LRE with EMS.

* *p* < 0.05 versus LRE

**p < 0.01 versus LRE.

† *p* < 0.05 versus EMS.

†† *p* < 0.01 versus EMS.

### 3.3 Changes in the CWST‐measured IC during the experimental session

The changes in reaction time and response accuracy across the three CWST task types during the LRE‐EMS, LRE, and EMS conditions are summarized in Table 2. Reaction times for both the congruent and neutral tasks did not show significant variation across any of the experimental conditions (Table 2). Reaction times in the incongruent task were reduced immediately after exercise and 15 min post‐exercise compared to baseline across all conditions (*p* = 0.005 at immediately after exercise; *p* < 0.001 at 15 min post‐exercise). The extent of changes in reaction time of incongruent task from baseline to immediately postexercise and from baseline to 15 min postexercise showed no significant difference among all the conditions (Table 3). No significant changes in response accuracy of the neutral or congruent tasks were observed under any of the experimental conditions (Table 2). For the LRE condition, compared with that at baseline, the response accuracy in the incongruent task decreased immediately after LRE (*p* = 0.019, *r* = 0.661). The extent of changes in response accuracy of incongruent task from baseline to immediately postexercise and from baseline to 15 min postexercise showed no significant difference among all the conditions (Table 3).

**Figure 2:**
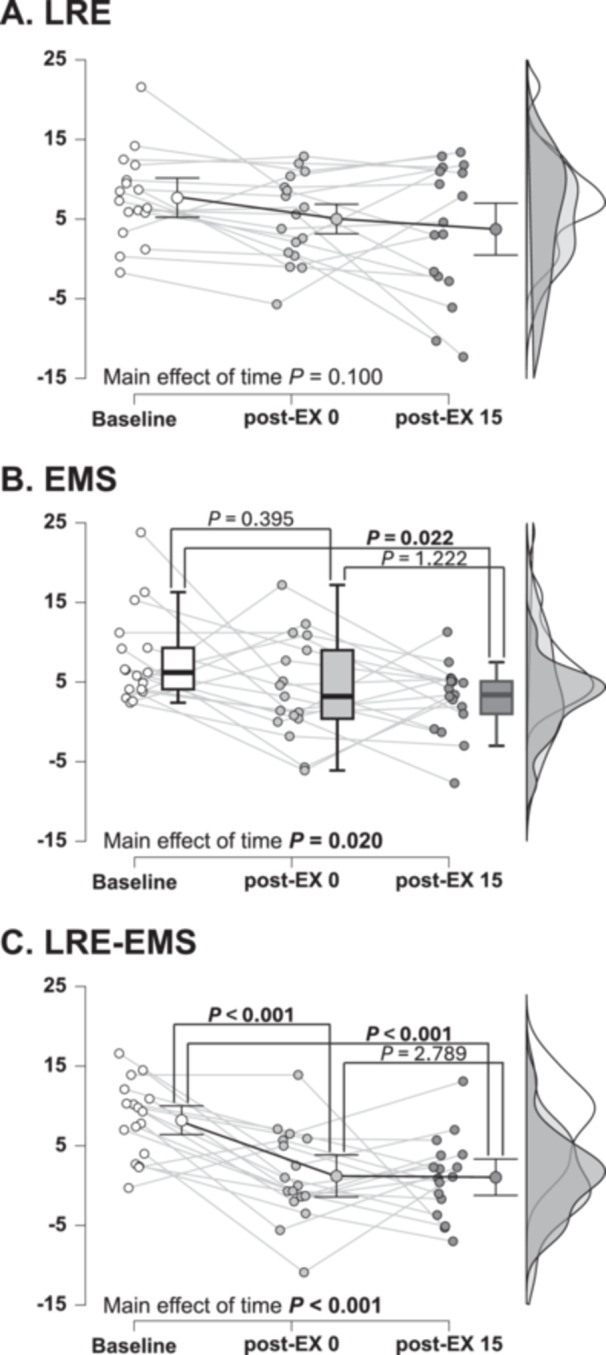
Changes in the reverse‐Stroop interference scores during the LRE‐EMS, LRE and EMS conditions. (A): Changes in reverse‐Stroop interference scores over time during LRE. (B): Changes in reverse‐Stroop interference scores over time during LRE. EMS. (C): Changes in reverse‐Stroop interference scores over time during LRE‐EMS. Data are presented as mean ± SD for normally distributed variables and as median (IQR) for non‐normally distributed variables. Statistical analyses were conducted separately within each condition, with one‐way repeated‐measures ANOVA applied when normality assumptions were met and Friedman tests used when they were not. When a significant main effect of time was observed, post hoc comparisons were performed using paired t tests or Wilcoxon signed‐rank tests with Bonferroni correction. The raincloud plots show the distribution of the reverse‐Stroop interference score. The circle plots represent individual data, and the box‐and‐ whisker plots represent the median values (IQR and max/min).

For the reverse‐Stroop interference score (Figure 2), significant main effects of time were observed for the EMS (*p* = 0.020, Figure 2b) and LRE‐EMS (*p* < 0.001, Figure 2c) conditions but not for the LRE condition (*p* = 0.100, Figure 2a). In the LRE‐EMS condition, the reverse‐Stroop interference score was lower immediately after exercise (*p* < 0.001, *d* = 1.078) and 15 min postexercise (*p* < 0.001, *d* = 1.363) than at baseline. In the EMS condition, the score did not significantly differ between immediately after exercise and baseline (*p* = 0.310, *r* = 0.395), but it was lower at 15 min postexercise than at baseline (*p* = 0.022, *r* = 0.652). These results indicate that IC was significantly greater immediately after LRE‐EMS than at baseline. Additionally, both the LRE‐EMS and EMS conditions demonstrated enhanced IC 15 min postexercise compared with baseline. No significant differences were found in the extent of change in reverse‐Stroop interference scores from baseline to immediately postexercise or baseline to 15 min postexercise across all conditions (Table 3).

### 3.4 Changes in psychological conditions for the CWST during the experimental session

Arousal was significantly greater immediately after exercise than at baseline for all the conditions (*p* = 0.006, *r* = 0.745 for LRE; *p* = 0.014, *r* = 0.686 for EMS; *p* = 0.003, *r* = 0.794 for LRE‐EMS). Arousal was significantly greater at 15 min postexercise than at baseline only in the LRE condition (*p* = 0.039, *r* = 0.603), not in the EMS (*p* = 0.098, *r* = 0.519) and LRE‐EMS condition (*p* = 0.250, *r* = 0.420). The extent of the change from baseline to immediately after exercise and from baseline to 15 min postexercise was not significantly different between the conditions. Compared with that at baseline, mental fatigue was significantly greater both immediately after exercise and 15 min postexercise for LRE (*p* = 0.010, *r* = 0.710 at post‐EX 0; *p* = 0.025, *r* = 0.640 at post‐EX 15) and LRE‐EMS (*p* = 0.006, *r* = 0.747 at post‐EX 0; *p* = 0.005, *r* = 0.758 at post‐EX 15). The extent of the change from baseline to immediately after exercise did not significantly differ between conditions. Changes in concentration and motivation were not observed under any of the experimental conditions (Table 2).

## 4 DISCUSSION

The main findings of this study are twofold. First, IC significantly improved immediately postexercise and remained significantly greater 15 min postexercise than at baseline under the LRE‐EMS condition, whereas no significant change in IC was observed in the LRE condition. Second, under the EMS condition, IC levels also demonstrated significant improvement at 15 min postexercise relative to baseline. These findings indicate that incorporating EMS into LRE can effectively enhance IC. Furthermore, the independent use of EMS also significantly increased postexercise IC levels.

The first major finding of this study is that incorporating EMS into LRE not only led to a greater increase in blood lactate levels but also, more importantly, resulted in a significant increase in IC, which was not observed for the LRE condition. Several factors may be involved in the potential mechanisms underlying the increase in IC observed under LRE‐EMS conditions. First, it has been suggested that lactate produced during exercise is preferentially used to meet the energy demands of neuronal activation during exercise and serves as a critical substrate supporting exercise‐induced IC (Hashimoto et al., 2018, 2021). Consistent with prior research findings using aerobic exercise (Watanabe et al., 2021), incorporating EMS into resistance exercise resulted in significantly elevated blood lactate levels compared with exercise alone. In the present research, incorporating EMS into LRE further increased postexercise blood lactate concentrations, which might partly contribute to the observed IC improvements under LRE‐EMS conditions. In addition, previous studies have suggested that EMS alone may enhance cerebral oxygenation, particularly in prefrontal regions associated with IC (Descollonges et al., 2024b). Furthermore, EMS has been shown to elicit cerebral oxygenation responses comparable to those observed during voluntary exercise, despite reduced voluntary muscle activation due to external assistance (Descollonges et al., 2026). Notably, improvements in certain Stroop task components have been observed only under EMS‐assisted voluntary exercise (Descollonges et al., 2025, 2026). Based on these findings, it is possible that the combined use of EMS facilitated neural activity, thereby contributing to the observed improvements in IC. However, it should be noted that these studies primarily assessed prefrontal responses during EMS or EMS‐assisted exercise, and it remains unclear whether EMS alone or in combination with exercise directly influences neural activity during cognitive task performance. Therefore, further research is needed to elucidate the mechanisms underlying the cognitive benefits of EMS alone and in combination with exercise.

In contrast to prior findings (Dora et al., 2021; Tsukamoto et al., 2017), no significant improvement in the IC was observed after LRE alone in this study. One possible explanation for this discrepancy is the variation in exercise protocols across studies. In the present study, the LRE intensity was set at 40% 1‐RM, which is higher than the 30% 1‐RM used in prior research (Dora et al., 2021). However, the total number of sets was reduced from six (Dora et al., 2021; Tsukamoto et al., 2017) to four, resulting in an overall lower exercise volume, which may have diminished the cognitive benefits observed for the LRE condition.

In contrast to a prior study (Watanabe et al., 2021) and our hypothesis, perceived exertion levels were slightly but significantly higher in the LRE‐EMS condition compared to the LRE condition. One possible explanation for this discrepancy lies in the EMS parameters, which may significantly influence participants' perceptions. Although the EMS parameters (e.g., frequency, pulse width) used in this study were based on prior research (Ando et al., 2021; Miyamoto et al., 2018), differences in intensity settings could have contributed to the significant increase in perceived exertion levels under the LRE‐EMS condition. Specifically, although the prior study (Watanabe et al., 2021) defined EMS intensity as the level at which participants could perform the exercise protocol without discomfort or stress, the present study set it as the maximum intensity at which participants could maintain proper form during leg extensions, even though this led to an increase in discomfort (Watanabe et al., 2021). Despite the significant increase in RPE observed when EMS was applied to LRE in this study, the higher intensity required—due to the shorter exercise duration—to elicit sufficient physiological responses, including elevated blood lactate levels, may have induced local muscle discomfort or nociceptive sensations and contributed to the heightened perceived exertion. Studies in stroke patients have shown that adding EMS to voluntary exercise can reduce perceived exertion while eliciting comparable physiological responses, such as cerebral blood flow and lactate production (Descollonges et al., 2025). In contrast, the present study included healthy young individuals without motor impairments, in whom EMS may provide limited functional assistance compared with clinical populations. Therefore, combining resistance exercise with EMS in populations with impaired motor function may enhance cognitive function while either reducing or not increasing perceived exertion.

In this study, EMS alone improved IC. One prior study reported results similar to those observed in this research (Descollonges et al., 2024b), demonstrating the potential of EMS alone to enhance cognitive function. Specifically, a prior study revealed improvements in short‐term memory and congruent Stroop task performance following a single EMS session. However, it did not report any improvements in IC. In contrast, the current study demonstrated a significant increase in IC after a single EMS session, thereby extending the scope of the documented benefits of EMS for executive function. Nevertheless, other prior studies have reported no significant improvements in cognitive performance immediately following EMS interventions (Ando et al., 2024b; Miyamoto et al., 2018). These prior studies examined only changes in cognitive function immediately postexercise without assessing sustained changes in cognitive function over time. Similar to the aforementioned studies, the present study did not observe cognitive improvements immediately post‐EMS intervention but identified significant improvements at 15 min postintervention. This finding suggests that the cognitive benefits of EMS may exhibit a delayed onset. Another critical aspect of this study is the shorter EMS duration compared with prior research. Although no studies have specifically investigated the optimal duration of EMS for enhancing cognitive function, evidence from active exercise studies indicates that exercise duration is a crucial factor influencing cognitive outcomes (Chang et al., 2015). This principle may also apply to EMS. In prior research, EMS protocols designed for active exercise, particularly aerobic exercise, often exceeded 20 min in duration (Ando et al., 2024b; Miyamoto et al., 2018). Although it might be too speculative, prolonged EMS durations might diminish cognitive benefits, whereas the shorter EMS duration in this study appears to have been more effective in enhancing cognitive function. However, as this study was conducted exclusively with healthy young male participants, caution is warranted in generalizing these findings to other populations, such as older adults, individuals with chronic health conditions, or females. Future research should investigate whether the cognitive benefits of EMS observed here can be replicated and extended across diverse demographic groups.

Previous studies have suggested that changes in arousal levels may influence exercise‐induced improvements in IC (Byun et al., 2014). Therefore, arousal was included in the present study as a potential modulator of IC performance. In addition, it has been proposed that EMS may enhance cognitive function through its effects on arousal (Descollonges et al., 2024a). In the present study, arousal significantly increased immediately after exercise in all conditions; however, improvements in IC were not observed in the LRE and EMS conditions. These findings suggest that the observed improvements in IC cannot be fully explained by changes in arousal alone, and that other physiological mechanisms may play a more prominent role.

## 5 LIMITATION

A limitation of the present study is the absence of a non‐exercise control condition, making it difficult to exclude the influence of learning effects on IC performance. However, participants underwent practice sessions prior to the experiment using the same familiarization protocol as our previous study (Tsukamoto et al., 2017), in which learning effects were demonstrated to be minimized. Notably, no improvement in IC was observed in the LRE condition despite repeated task exposure, suggesting that the observed improvements in the EMS and LRE + EMS conditions are unlikely to be explained solely by learning effects.

Another limitation is that blood lactate was measured immediately after exercise, which may not reflect peak lactate concentrations that typically occur several minutes post‐exercise (Freund and Zouloumian, 1981). Therefore, the present measurements may have underestimated peak lactate responses. However, as all measurements were conducted at the same time points across conditions, and significant increases in blood lactate were observed after the intervention in all conditions, this limitation is unlikely to substantially affect the interpretation of the results.

## 6 PERSPECTIVE

This study offers new insight into the potential of a practical strategy for cognitive improvement. Both LRE‐EMS and EMS elicited cognitive improvements following only 260 s of intervention, and prior evidence suggests that the cognitive benefits of regular exercise result from repeated acute improvements in cognitive performance triggered by such individual sessions (Hashimoto et al., 2021; Voss et al., 2020). Given that lack of time is a major barrier to consistent physical activity, these findings underscore the promise of time‐efficient, accessible interventions—particularly EMS, which involves passive muscle stimulation and may be suitable for individuals with physical limitations. Together, these findings position LRE‐EMS and EMS as promising, accessible strategies for cognitive improvement, meriting further investigation into their long‐term effects. Notably, the observation that EMS alone improved cognitive function suggests that skeletal muscle contraction, even in the absence of voluntary exertion, may be sufficient to induce cognitive benefits. Future studies elucidating the physiological mechanisms underlying EMS‐induced cognitive improvement will be essential—not only to inform the development of effective and scalable EMS‐based interventions, but also to contribute to a broader understanding of how muscular contraction can modulate cognitive function. Such insights could advance both preventative and therapeutic strategies for mitigating cognitive decline across aging, clinical, and sedentary populations.

## 7 CONCLUSION

Integrating EMS into LRE effectively enhanced cognitive function, but increased RPE still existed. Also, EMS alone demonstrated unique advantages by improving IC without active exercise, making it suitable for individuals with physical limitations. These results highlight LRE‐EMS and EMS as effective and accessible strategies for cognitive improvement. Future research should investigate the long‐term impacts and elucidate the mechanisms underlying these cognitive improvements.

## AUTHOR CONTRIBUTIONS

Kento Dora, Su Yang, and Takeshi Hashimoto conceived and designed the research. Kento Dora, Su Yang, I. Wayan Yuuki, Kousei Tachi, Kaito Hashimoto, and Teppei Matsumura conducted the experiments. Kento Dora and Su Yang collected the data and performed statistical analyses. Kento Dora, Su Yang, I. Wayan Yuuki, Kousei Tachi, Kaito Hashimoto, Teppei Matsumura, Naokazu Miyamoto, and Takeshi Hashimoto interpreted the results. Kento Dora prepared the figures. Kento Dora and Su Yang drafted the manuscript, and all authors reviewed and revised it. All authors have read and approved the final version of the manuscript and agree to be accountable for all aspects of the work, ensuring that any questions related to the accuracy or integrity of any part of the work are appropriately investigated and resolved.

## CONFLICT OF INTEREST STATEMENT

The authors declare no conflicts of interest.

## ETHICS STATEMENT

The study was conducted in accordance with the Declaration of Helsinki, and all procedures were approved by the Ethics Committee of Ritsumeikan University (BKC‐LSMH‐2023‐100‐1).

## CONSENT FOR PUBLICATION

The participants cannot be individually identified from the data published in this manuscript. The participants were made aware of the intent to publish these data when providing informed consent.

## PATIENT CONSENT STATEMENT

The participants provided written informed consent before participation under the principles of the Declaration of Helsinki. The participants cannot be individually identified from the data published in this manuscript. The participants were made aware of the intent to publish these data when providing informed consent.

## CLINICAL TRIAL REGISTRATION

The study was registered in the University Hospital Medical Information Network Clinical Trials Registry as UMIN000057559.

## Supporting information

Supporting File 1.

## Data Availability

Scandinavian Journal of Medicine & Science in Sports (SMS). The datasets generated and analyzed during the current study are available from the corresponding author upon reasonable request.
