# Supplementary material for: The combination of low‐intensity resistance exercise and electrical muscle stimulation effectively enhances executive function in men
Source: Clin Physiol Funct Imaging. 2026 Jun 23;46(4):e70075. doi: 10.1111/cpf.70075 (PMC13291766; doi:10.1111/cpf.70075)
Supplement: Supplementary file 1 — Supporting File 1. [file CPF-46-0-s001.docx]

**Supplemental Table 1. Baseline states**

|  | Condition | | | one-way ANOVA  or  Friedman test |
| --- | --- | --- | --- | --- |
|  | LRE | EMS | LRE-EMS | *p* value |
| ***Blood metabolites*** |  |  |  |  |
| Glucose (mg/dl) | 92 ± 11 | 89 ± 7 | 91 ± 8 | 0.488 |
| Lactate (mM) | 1.4 (1.0-2.3) | 1.3 (0.9-2.5) | 1.4 (1.0-2.4) | 0.554 |
| ***Color-word Stroop tasks*** |  |  |  |  |
| Reaction time (msec) |  |  |  |  |
| Congruent task | 11705 ± 1620 | 12234 ± 2520 | 12175 ± 2223 | 0.334 |
| Neutral task | 12545 ± 2142 | 12457 ± 2384 | 12311 ± 2046 | 0.588 |
| Incongruent task | 13499 ± 2464 | 13425 ± 2530 | 13402 ± 2412 | 0.906 |
| Response accuracy (%) |  |  |  |  |
| Congruent task | 99 (92-100) | 96 (86-100) | 97 (92-100) | 0.414 |
| Neutral task | 100 (93-100) | 100 (83-100) | 99 (88-100) | 0.096 |
| Incongruent task | 99 (89-100) | 99 (92-100) | 99 (94-100) | 0.052 |
| Interference scores (%) | 7.3 (-1.7-21.6) | 6.2 (2.4-23.8) | 9.3 (-0.3-16.6) | 0.662 |
| ***Psychological states*** |  |  |  |  |
| Felt arousal scale, 1-6 |  |  |  |  |
| Arousal | 3 (2-4) | 3 (2-5) | 3 (2-4) | 0.625 |
| Visual analog scales, 0-100 mm |  |  |  |  |
| Mental fatigue | 13 (1-61) | 17 (1-79) | 13 (1-72) | 0.230 |

Values are mean ± SD or median (IQR). The *p*-values shown in the table represent the results of the comparison between LRE-EMS, LRE and

EMS conditions in baseline states by one-way ANOVA or Friedman test.
